# Development of Multilayer Magnetic Janus Sub-Micrometric Particles for Lipase Catalysis in Pickering Emulsion

**DOI:** 10.3390/molecules30112429

**Published:** 2025-05-31

**Authors:** Wei Wang, Xiangyao Chen, Wen-Can Huang, Simiao Di, Jie Luo

**Affiliations:** 1 State Key Laboratory of Marine Food Processing and Safety Control, College of Food Science and Engineering, Ocean University of China, Qingdao 266404, China; 2 Marine Science Research Institute of Shandong Province, Qingdao 266104, China; 3 Qingdao Key Laboratory of Food Biotechnology, Qingdao 266404, China; 4 Key Laboratory of Biological Processing of Aquatic Products, China National Light Industry, Qingdao 266404, China

**Keywords:** lipase, Pickering emulsion, immobilization, enzyme immobilization

## Abstract

This study presents a multilayer magnetic Janus sub-micrometric particle (MMJSP) as a nanoreactor for lipase catalysis. The core of the nanoparticle is constructed from a core-shell Fe_3_O_4_@SiO_2_ framework, which serves as a precursor for the sequential amino and aldehyde modifications using 3-aminopropyltriethoxysilane and benzaldehyde. Following localized etching and subsequent modification with N,N-dimethyldodecylamine, a Janus nanoparticle with distinct hydrophilic and hydrophobic domains is synthesized. The resulting MMJSP demonstrates a stable attachment to the reaction interface and significantly enhances lipase performance, exhibiting 1.4-fold and 1.6-fold enhancements in activity after immobilization during 1 h hydrolysis and 24 h esterification reactions, respectively. Additionally, the storage stability of the immobilized lipase is improved by 100% over a period of 30 days. Reusability assessments reveal that the immobilized enzyme retains 80.7% activity after 10 cycles of esterification and 80.6% after 50 cycles of hydrolysis, with the magnetic properties allowing for rapid separation and recovery of the immobilized enzyme.

## 1. Introduction

Lipases, also referred to as triacylglycerol hydrolases, serve as effective biocatalysts for a variety of hydrolysis and synthesis reactions [1,2,3]. These enzymes have gained prominence in various industries, including food production, healthcare, chemical manufacturing, environmental remediation, biodiesel production, and biosensing [4]. As interfacial enzymes, most lipases feature a distinctive “lid” structure, characterized as an amphiphilic α-helical loop that conceals the active site within a catalytic triad made up of Ser-His-Asp/Glu [5,6,7]. The lid structure typically remains in a closed conformation in a homogeneous saline solution, effectively shielding the active center from the hydrophilic environment. When lipase acts at the hydrophilic–hydrophobic interface, the lid undergoes a conformational change, allowing hydrophobic substrates to access the active site [8]. This process, known as “interfacial activation”, highlights the necessity of the water–oil interface and the associated conformational adjustments for effective lipase catalysis [9,10].

Biphasic systems have emerged as a powerful strategy to enhance the efficiency and selectivity of lipase-catalyzed reactions [11]. These systems typically consist of two immiscible liquid phases—an organic solvent and an aqueous phase—which provide an environment conducive to the partitioning of substrates and products based on their hydrophobicity [12,13]. This phase separation is particularly beneficial for lipases, whose hydrophobic characteristics facilitate improved interactions with non-polar substrates residing in the organic phase. Additionally, biphasic systems can mitigate substrate inhibition and enhance enzyme stability, as the presence of an organic solvent often protects the enzyme from denaturation caused by high substrate concentrations. The implementation of biphasic configurations also simplifies product recovery, thus increasing the overall efficiency of the catalytic process. Nonetheless, the use of free lipase for hydrolysis at the oil–water interface presents several challenges, including the tendency for enzyme molecules to disperse into the aqueous phase, reduced reusability, diminished activity due to instability, and the potential for lipase–lipase dimer formation through hydrophobic interactions involving the open conformation of the enzyme [14]. However, biphasic reaction systems are limited by the finite contact area.

Pickering emulsions are stabilized by ultrafine solid particles, the particles precisely positioned at the interface of the aqueous or liquid–liquid phases [15,16,17]. The contact between the oil and water phases creates a vast specific surface area, which significantly enhances catalytic efficiency [18]. In recent years, the interfacial enzyme catalysis system based on Pickering emulsions has found extensive applications in the enhancement of lipid flavor, high-value utilization of high-acid-value oils, and incorporation of food additives [19,20,21]. Constructing stimuli-responsive Pickering emulsion enzymatic reaction systems for rapid and efficient separation of enzymes and catalytic products is a significant development direction for Pickering emulsion biocatalytic systems. Magnetic nanomaterials offer enhanced mass transfer and rapid magnetic recovery in Pickering emulsion biocatalytic systems [22,23]. Notably, Janus particles are characterized by having two or more sides with distinct physicochemical properties, the unique structure enables them to exhibit superior emulsion stabilization performance. Meanwhile, it had gained significant traction as an innovative carrier for lipase immobilization. These colloidal particles possess two distinct surfaces with differing chemical or physical properties, enabling selective interactions with both hydrophilic and hydrophobic environments [24,25]. This structural duality makes Janus particles particularly well-suited for incorporation into biphasic systems [26,27]. Their tailored surface characteristics can facilitate improved enzyme-substrate interactions, leading to increased catalytic efficiency while also enhancing the stability of the enzyme under various reaction conditions. Enzyme immobilization using Janus particles capitalizes on their asymmetric surface topology to enhance interfacial activity. The amphiphilic nature enables the stabilization of the Pickering Emulsion while maintaining catalytic pocket accessibility [28,29].

In this study, a novel multilayer magnetic Janus sub-micrometric particle (MMJSP) was developed as a carrier for lipase immobilization, utilizing a core-shell structured Fe_3_O_4_@SiO_2_ as the precursor. Fe_3_O_4_, as the core and the surface of silicon dioxide, is rich in active groups, which can provide abundant sites for the covalent immobilization of enzymes, utilizing a core-shell structured Fe_3_O_4_@SiO_2_ as the precursor. Following amino and aldehyde modifications, a localized etching process was employed to further refine the structure, after which the etched regions underwent hydrophobic modification, resulting in Janus sub-micrometric particles with distinct hydrophilic and hydrophobic domains. Through Schiff base formation between aldehyde-modified Janus particles and lipase amino groups, we achieve covalent enzyme immobilization while leveraging the particles’ amphiphilicity for emulsion stabilization, thereby enabling efficient interfacial catalysis.

## 2. Materials and Methods

### 2.1. Materials

Lipase B were purchased from Beijing Cliscent Technology Co., Ltd. (Beijing, China) Solid paraffin (mp 58–62 °C), N,N-dimethyl-dodecylamine (DDA), Nile red (≥95.0%), palm acid p-nitrophenyl ester, isopropanol, dimethyl sulfoxide (DMSO), palm acid p-nitrophenol, 3-aminopropyltriethoxysilane (APTES), fluorescein isothiocyanate (FITC), tetraethyl orthosilicate (TEOS), hexanoic acid, 1-hexanol, and hexane were purchased from Aladdin Biochemical Technology Co., Ltd. (Shanghai, China). Iron(II) chloride, iron(III) chloride, ammonia, and anhydrous ethanol were purchased from Macklin Biochemical Co., Ltd. (Shanghai, China).

### 2.2. Synthesis of Paramagnetic Core-Shell Structured Nanobeads

Initially, 3.2 g of Ferrous chloride and 10.8 g of ferric chloride are dissolved in 300 mL of ultrapure water. The solution is stirred using a magnetic stirrer under a nitrogen atmosphere for 5 min to ensure complete dissolution. Following this, 4 mL of concentrated ammonia solution (25% NH_3_ H_2_O) is added, resulting in a rapid color change to black. The reaction is continued under nitrogen for an additional 30 min. Upon completion, nanosized Fe_3_O_4_ is separated using an external magnetic field, followed by washing with anhydrous ethanol and ultrapure water and then vacuum-drying at room temperature [30,31].

Then, 1 g of Fe_3_O_4_ particles is dispersed in 50 mL of a 20% sodium citrate solution and subjected to sonication for 30 min, followed by thorough washing with ethanol and water. The treated Fe_3_O_4_ particles, 15 mL of water, and 4.5 mL of NH_3_·H_2_O are added to 180 mL of anhydrous ethanol, and stirring is performed for 1 h. Afterward, 10 mL of TEOS (28%) is introduced, and the reaction is allowed to proceed for 8 h. The final mixture is washed three times with anhydrous ethanol and water to yield Fe_3_O_4_@SiO_2_ (Fe_3_O_4_ nanoparticles covered with silica) particles [32].

### 2.3. Synthesis of Multilayer Magnetic Janus Sub-Micrometric Particles

Initially, 1 g of Fe_3_O_4_@SiO_2_ particles and 1000 μL of 3-aminopropyltriethoxysilane (APTES) were added to 100 mL of toluene, and the reaction was refluxed at 80 °C for 8 h. After cooling to room temperature, the mixture was washed three times with toluene to obtain Fe_3_O_4_@SiO_2_-NH_2_ particles. These particles, along with 500 mg of benzaldehyde and 50 μL of glacial acetic acid, were added to 300 mL of ethanol, and the solution was reacted at room temperature for 12 h. Following three washes with ethanol and water, benzaldehyde-modified Fe_3_O_4_@SiO_2_ particles were obtained [33].

At 70 °C, 1 g of benzaldehyde-modified Fe_3_O_4_@SiO_2_ particles and 10 g of paraffin were added to 15 mL of water, and the mixture was stirred at 8000 r/min for 3 min by homogenizer. Once the emulsion cooled to room temperature, the paraffin emulsion solidified. The emulsion droplets were added to 2 mL of a 5% *w/v* NH_4_F aqueous solution and etched for 1 h. Subsequently, tetrahydrofuran (THF) was added for three washes to remove the paraffin. The etched sub-micrometric particles were dispersed in 100 mL of THF, and 1000 μL of N,N-dimethyldodecylamine (DDA) was added for a sealed reaction at room temperature for 12 h.

### 2.4. Characterization

The chemical structure of the samples was analyzed using a Fourier Transform Infrared (FT-IR) spectrometer (Nicolet iS10, Thermo Scientific, Waltham, MA, USA), scanning the range from 4000 cm^−1^ to 400 cm^−1^. The surface morphology of the samples was examined using a Scanning Transmission Electron Microscope (Quanta 200, FEI Tecnai, Ann Arbor, MI, USA) in High-Angle Annular Dark Field (HAADF) mode with an acceleration voltage of 120 kV. The elemental composition of the sample surfaces was determined using Energy Dispersive X-ray (EDX) mapping with a Transmission Electron Microscope (Mic JEM-1200EX, JEOL, Akishima, Japan) at a working voltage of 200 kV. The particle sizes were determined using a Zetasizer Nano-ZS (ZS90, Malvern, England). A 10 µL sample of Pickering emulsion was placed on a glass slide, covered with a coverslip to stabilize the emulsion, and observed under a bright field of an electric fluorescence microscope (Nikon, Minato City, Japan). Subsequently, the emulsion morphology was observed under an excitation wavelength of 488 nm using fluorescein isothiocyanate (FITC)-labeled lipase. The contact angles were measured using a contact angle analyzer (DSAHT17C, KRUSS, Hamburg, Germany). based on the sessile drop method at 25 °C.

### 2.5. Enzyme Immobilization

A solution of lipase B (100 μL) was added to 7.4 mL of phosphate buffer (100 mM, pH 7.4). The enzyme solution was mixed with 100 mg of magnetic Janus particles in 7.5 mL of n-hexane, and homogenization was performed at 10,000 rpm for 2 min.

### 2.6. Enzyme Activity Assay

p-Nitrophenyl palmitate (pNPP) was used as the substrate for the lipase hydrolysis reaction. One unit of lipase activity (U) was defined as the amount of enzyme required to hydrolyze pNPP to produce 1 μmol of p-nitrophenol (pNP) per min. First, pNPP (20 mM) was dissolved in a mixed solvent of isopropanol/DMSO (1:1). The immobilized enzyme was then dispersed in 450 μL of phosphate buffer (100 mM, pH 7.4) and incubated in a water bath at 37 °C for 5 min. Subsequently, 50 μL of the prepared pNPP (20 mM) was added and allowed to react for 5 min. After the reaction, the amount of pNP was measured.

The esterification reaction of the lipase was assessed by preparing hexyl hexanoate using hexanol and hexanoic acid as substrates in a Pickering emulsion system. The 1 mM of hexanol and 1 mM of hexanoic acid were dissolved in 3 mL of n-hexane, 100 mg of immobilized enzyme was dispersed in 1 mL of phosphate buffer (pH 7.4), and the mixture was homogenized at 12,000 rpm for 2 min to form the Pickering emulsion. The emulsion was incubated at 37 °C for 10 min, and the immobilized enzyme was separated using an external magnetic field. The 10 μL of the upper organic phase was then analyzed for hexyl hexanoate content using gas chromatography (GC-2010, Shimadzu, Kyoto, Japan). The GC conditions were as follows: FID detector, INNOWAX polar column (30.0 m length, 0.53 mm inner diameter, 1.00 μm film thickness), with an initial temperature of 80 °C held for 0.5 min, ramping at 20 °C/min to 170 °C, and then increasing at 5 °C/min to 200 °C.

### 2.7. Catalytic Efficiency Testing

The hydrolysis and esterification reactions were catalyzed using the same amount of free and immobilized enzymes. The reactions were conducted for 6 h, during which samples were continuously taken to measure the product yield. The reactions were performed at pH 7.5 and 37 °C.

### 2.8. Stability and Reusability Testing of Immobilized Enzymes

To assess pH stability, both free and immobilized enzymes were incubated at room temperature in buffer solutions with pH values ranging from 2.0 to 10.0 for 180 min, and their activities were measured. For temperature stability, both types of enzymes were incubated at temperatures between 30 °C and 70 °C for 180 min, followed by activity measurement. To evaluate storage stability, both free and immobilized enzymes were stored at room temperature for 28 d, with multiple samples taken to measure activity. To determine reusability, the immobilized enzyme was subjected to hydrolysis and esterification reactions ten times, with enzyme activity measured for each reaction.

## 3. Results and Discussion

### 3.1. Characterization of Multilayer Magnetic Janus Sub-Micrometric Particles

**Figure 1:**
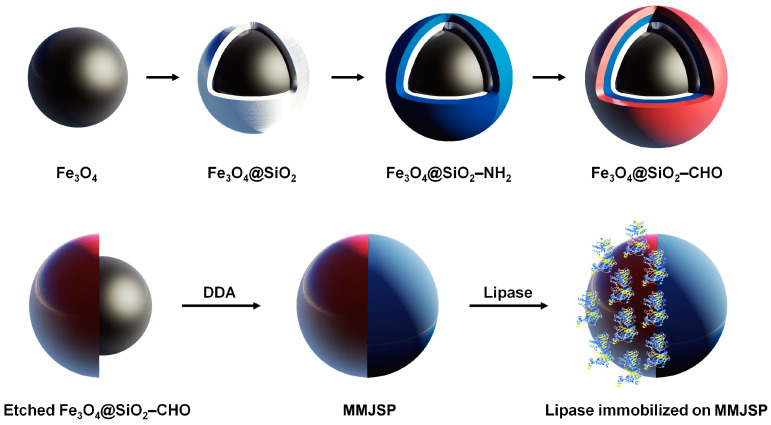
Schematic diagram of the preparation of MMJSP-immobilized lipase.

**Figure 2:**
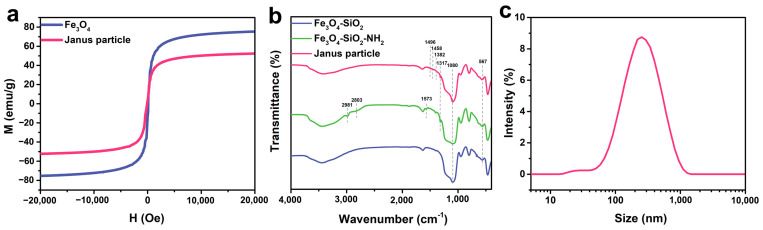
Characterization of the samples: (**a**) Magnetization curves obtained by a vibrating sample magnetometer for Fe₃O₄ and MMJSP. (**b**) FTIR spectra of Fe₃O₄@SiO₂, Fe₃O₄@SiO₂-NH₂, and MMJSP. (**c**) Size distribution profile of the MMJSP.

Figure 1 illustrates the process of synthesizing MMJSP and immobilizing lipase. Paramagnetic spherical Fe_3_O_4_ particles were prepared through chemical co-precipitation. The magnetization curve obtained from a vibrating sample magnetometer indicated that the magnetic intensity of the Fe_3_O_4_ particles was 75.3 emu/g, with no residual magnetism detected from the hysteresis loop, confirming the paramagnetic nature of the Fe_3_O_4_ particles (Figure 2a). After the preparation of the Fe_3_O_4_ particles, SiO_2_ was coated onto their surface via a sol-gel reaction, resulting in core-shell structured Fe_3_O_4_@SiO_2_ particles. Hysteresis loop results indicated that the magnetization of the Fe_3_O_4_@SiO_2_ particles was 52.3 emu/g, confirming their continued paramagnetic properties (Figure 2a).

The surface of the Fe_3_O_4_@SiO_2_ particles was hydrophilic due to the presence of silanol groups, leading to instability at the interface between phases, which rendered the Pickering emulsion formed with these particles as solid emulsifiers unstable. Therefore, the surface was aminated using APTES, followed by the introduction of benzaldehyde through a Schiff base reaction with the amine. FT-IR revealed a characteristic peak at 567 cm^−1^ corresponding to Fe-O vibrations and a peak at 1080 cm^−1^ attributed to the asymmetric stretching of Si-O-Si (Figure 2b). Following APTES modification, peaks at 2981 cm^−1^ and 2803 cm^−1^ were observed, corresponding to symmetric and asymmetric stretching vibrations of -CH_2_, respectively. An absorption peak at 1573 cm^−1^ was associated with -NH_2_ in-plane bending vibrations, and a peak at 1317 cm^−1^ corresponded to C-N stretching vibrations. After the reaction with benzaldehyde, absorption peaks in the range of 1608 to 1382 cm^−1^ were noted, corresponding to benzene ring skeletal vibrations, while a peak at 725 cm^−1^ was attributed to aromatic C-H stretching vibrations, confirming the successful modification with benzaldehyde.

**Figure 3:**
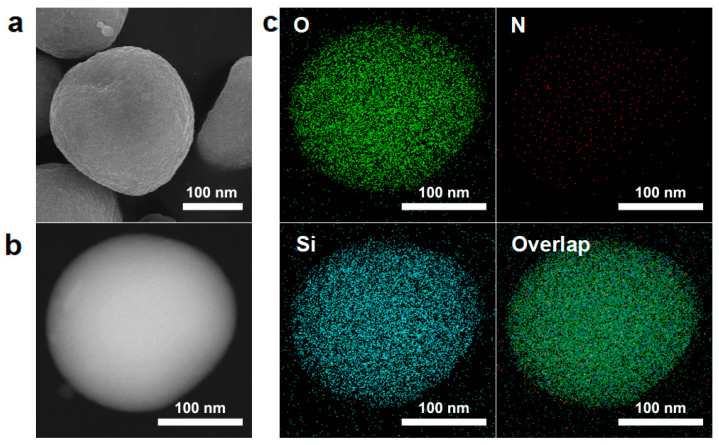
Morphological characteristics of the MMJSP: (**a**) SEM image, (**b**) STEM image, and (**c**) EDX mapping analysis of the MMJSP.

One side of the sub-micrometric particles embedded in paraffin could be selectively protected, while the other side was exposed to the aqueous phase. By selectively etching the side facing the aqueous phase, an asymmetric Janus structure was formed. Paraffin droplets were dispersed in an NH_4_F aqueous solution to dissolve the exposed silica, followed by the removal of paraffin using tetrahydrofuran (THF) to obtain the magnetic Janus sub-micrometric particles. The size of the MMJSP is approximately 200 nm (Figure 2c). SEM and HAADF images displayed the Janus particles (Figure 3a,b), and energy-dispersive X-ray (EDX) analysis revealed the asymmetric elemental distribution, indicating the successful preparation of magnetic Janus sub-micrometric particles (Figure 3c).

### 3.2. Characterization of Pickering Emulsions

The characteristics of the emulsion, including stability and droplet size, are critical for interfacial reactions and directly affect the reaction rate of lipase. As shown in Figure S1a, the emulsion droplets were spherical, with diameters ranging from 5 to 30 μm. To confirm the location of the lipase, it was labeled with fluorescein isothiocyanate (FITC). A distinct fluorescence ring was observed at the emulsion interface, confirming that the lipase was uniformly distributed at the oil–water interface (Figure S1b). Furthermore, the rapid demulsification response of the emulsion is significant for the recovery of immobilized lipase. As illustrated in Figure S2, under the influence of an external magnetic field, the Pickering emulsion could be rapidly demulsified, and after multiple repeated reactions, no significant changes in emulsion properties were observed.

### 3.3. Catalytic Performance Testing

**Figure 4:**
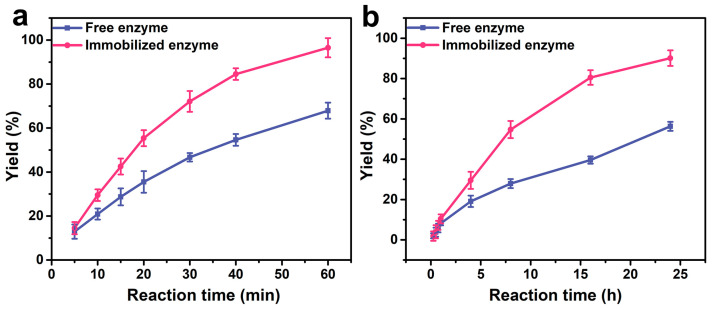
Time courses of (**a**) the hydrolysis and (**b**) the esterification reactions using free lipase and MMJSP-immobilized lipase.

To investigate the effect of magnetic Janus sub-micrometric particles as carriers on lipase activity, the catalytic efficiency of free enzymes and immobilized enzymes was compared. In the hydrolysis reaction, after 1 h, the immobilized enzyme hydrolyzed 96.5% of pNPP, which was 1.4 times that of the free enzyme (Figure 4a). In the esterification reaction, after 24 h, the conversion rate of hexanol and hexanoic acid catalyzed by the immobilized enzyme reached 90.1%, 1.6 times higher than that of the free enzyme (Figure 4b). The catalytic efficiency of the Pickering emulsion catalytic system is improved compared to the traditional free enzyme biphasic system. This is attributed to the fact that the Janus particles can precisely control the positioning of lipase at the oil/water interface of the emulsion droplets. This not only shortens the diffusion distance of substrate molecules between the two phases but also reduces the mass transfer resistance, thereby achieving higher catalytic efficiency.

Enzymatic reaction kinetics revealed that the Km values of both the immobilized and free enzymes were 0.17 × 10^−3^ mol/L·min, indicating that the substrate affinity of the enzyme did not change significantly before and after immobilization. Furthermore, the Vmax values for the free and immobilized enzymes were 0.41 mol/L and 0.44 mol/L, respectively, indicating a significant increase in the reaction efficiency of the immobilized enzyme when magnetic Janus sub-micrometric particles were used as micro-reactors (Table S1).

### 3.4. Stability Testing

**Figure 5:**
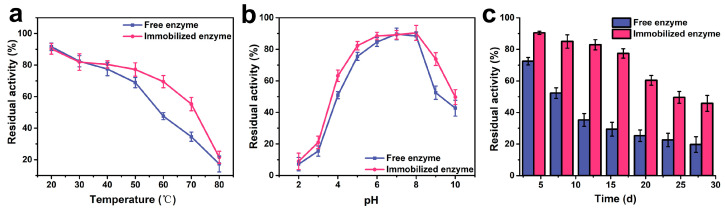
(**a**) Thermal stability, (**b**) pH stability, and (**c**) storage stability of the free and immobilized enzymes.

In addition to enzyme activity, stability is another important indicator for evaluating enzymes. Regarding thermal stability, no significant differences in stability were observed between free lipase and immobilized lipase at lower temperatures (Figure 5a). At 30 °C, the residual enzyme activities of both the free and immobilized enzymes were 82.4% and 81.9%, respectively. However, at higher temperatures, the thermal stability of the immobilized lipase was significantly greater than that of the free enzyme, with the residual enzyme activities of the immobilized lipase at 60 °C and 70 °C being 45.7% and 59.8% higher than those of the free enzyme, respectively. In terms of pH stability, the residual enzyme activities of the immobilized lipase were higher than those of the free enzyme under different pH conditions, with residual activities at pH 3.0 and pH 9.0 being 35.2% and 40.7% greater than those of the free enzyme, respectively (Figure 5b). For storage stability, a clear decline in enzyme activity was observed for the free enzyme over time, with less than 20% activity remaining after 30 d. In contrast, the immobilized enzyme exhibited a gradual decline in activity, maintaining 80% of its initial activity after 15 d and over 40% after 30 d, demonstrating enhanced storage stability (Figure 5c). The covalent conjugation of enzymes to carrier matrices effectively shields the biocatalysts from environmental perturbations, particularly thermal fluctuations and pH variations. Notably, lipase, which possesses interfacial activation characteristics, has its active site covered by a hydrophobic lid. When immobilized on a hydrophobic carrier, the active site of the lipase can be opened. The lipase can adsorb onto the hydrophobic side of Janus particles through hydrophobic interactions, thereby enhancing enzyme activity.

### 3.5. Reusability Testing

**Figure 6:**
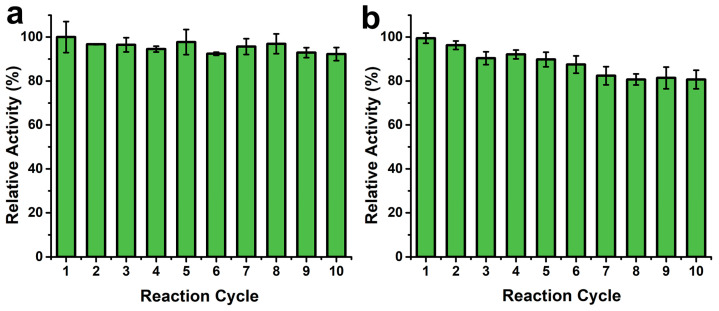
Reusability of the immobilized enzymes. (**a**) hydrolysis reactions, (**b**) esterification reactions.

Reusability is one of the most critical characteristics of immobilized enzymes. To investigate reusability, the immobilized enzyme was subjected to repeated catalysis in hydrolysis and esterification reactions for ten cycles. As shown in Figure 6, the immobilized enzyme demonstrated good reusability. In the hydrolysis reaction, the enzyme activity of the immobilized enzyme remained at 97.7% of the initial activity after five cycles, and 92.2% of the initial activity was maintained after 10 cycles (Figure 6a). In the esterification reaction, after five cycles, the enzyme activity of the immobilized lipase remained at 89.8% of the initial activity, while after 10 cycles, 80.7% of the initial activity was retained (Figure 6b).

To further investigate the reusability of lipase immobilized on MMJSP, the immobilized lipase was reused 50 times in hydrolysis reactions using a conventional two-phase system. The results indicated that after 50 cycles of reuse, the enzyme activity remained above 80%, demonstrating good reusability of the immobilized lipase in this reaction system (Figure S6). 

## 4. Conclusions

This study successfully utilized novel paramagnetic Janus particles as carriers to achieve enzyme immobilization and the preparation of an efficient Pickering emulsion catalytic system. Through precise surface modification, the Janus particles exhibited amphiphilic properties, enabling the stabilization of Pickering emulsions and the effective localization of enzyme molecules at the oil–water interface, thereby optimizing the interaction between enzymes and substrates. Furthermore, the magnetic responsiveness of the Janus particles allowed the Pickering emulsion catalytic system to be efficiently recovered under an external magnetic field, simplifying the recycling process of the immobilized enzymes and endowing them with high reusability. After 10 cycles of hydrolysis and esterification reactions, the Pickering emulsion catalytic system retained 80.7% and 87.9% of its initial activity, respectively. Compared to traditional free enzyme systems, the immobilized enzymes demonstrated superior stability under high temperatures and a wide pH range. In catalytic performance tests for esterification and hydrolysis reactions, the yields of the Pickering emulsion catalytic system were 1.6 times and 1.4 times higher than those of free enzymes, respectively, validating its potential for industrial applications.

## Data Availability

The raw data supporting the conclusions of this article will be made available by the authors on request.

## Supplementary Materials

The following supporting information can be downloaded at https://www.mdpi.com/article/10.3390/molecules30112429/s1: Materials, microscopic images of Pickering emulsions formed by MMJSP-immobilized lipase, photographs of the MMJSP immobilized lipase dispersed in a solution and the MMJSP immobilized lipase attracted by external magnetic fields, enzyme activity of MMJSP -immobilized lipase after 50 repeated uses in hydrolysis reactions, and kinetic parameters of the free and MMJSP-immobilized lipase.
