# Supplementary material for: Development of Multilayer Magnetic Janus Sub-Micrometric Particles for Lipase Catalysis in Pickering Emulsion
Source: Molecules. 2025 May 31;30(11):2429. doi: 10.3390/molecules30112429 (PMC12157209; doi:10.3390/molecules30112429)
Supplement: Supplementary file 1 [file molecules-30-02429-s001.zip › molecules-3493600-supplementary.pdf]

Supplementary for

Development of Multilayer Magnetic Janus Sub-micrometric Particles for Lipase Catalysis in Pickering Emulsion

Characterization

The chemical structure of the samples was analyzed using a Fourier Transform Infrared (FT-IR) spectrometer (Nicolet iS10, Thermo Scientific, Waltham, MA, USA), scanning the range from 4000 cm^{-1} to 400 cm^{-1}. The surface morphology of the samples was examined using a Scanning Transmission Electron Microscope (Quanta 200, FEI Tecnai, Ann Arbor, MI, USA) in High-Angle Annular Dark Field (HAADF) mode with an acceleration voltage of 120 kV. The elemental composition of the sample surfaces was determined using Energy Dispersive X-ray (EDX) mapping with a Transmission Electron Microscope (Mic JEM-1200EX, JEOL, Akishima, Japan) at a working voltage of 200 kV. The particle sizes were determined using a Zetasizer Nano-ZS (ZS90, Malvern, England). A 10 μL sample of Pickering emulsion was placed on a glass slide, covered with a coverslip to stabilize the emulsion, and observed under a bright field of an electric fluorescence microscope (Nikon, Minato City, Japan). Subsequently, the emulsion morphology was observed under an excitation wavelength of 488 nm using fluorescein isothiocyanate (FITC)-labeled lipase.The contact angles were measured using a contact angle analyzer (DSAHT17C, KRUSS, Hamburg, Germany). based on the sessile drop method at 25 °C.

Results

Characterization of Pickering Emulsions

The characteristics of the emulsion, including stability and droplet size, are critical for interfacial reactions and directly affect the reaction rate of lipase. As shown in Figure S1a, the emulsion droplets were spherical, with diameters ranging from 5 to 30 μm. To confirm the location of the lipase, it was labeled with fluorescein isothiocyanate (FITC). A distinct fluorescence ring was observed at the emulsion interface, confirming that the lipase was uniformly distributed at the oil-water interface (Figure S1b). Furthermore, the rapid demulsification response of the emulsion is significant for the recovery of

immobilized lipase. As illustrated in Figure S2, under the influence of an external magnetic field, the Pickering emulsion could be rapidly demulsified, and after multiple repeated reactions, no significant changes in emulsion properties were observed.

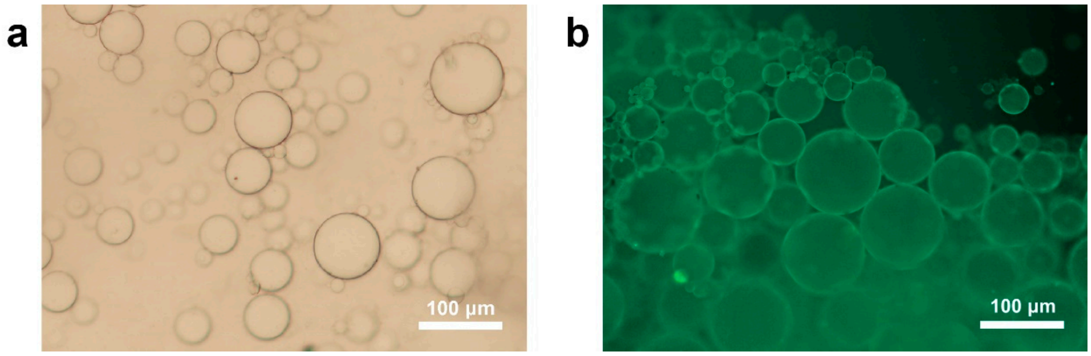

Figure S1. Microscopic images of Pickering emulsions formed by MMJSP-immobilized lipase.

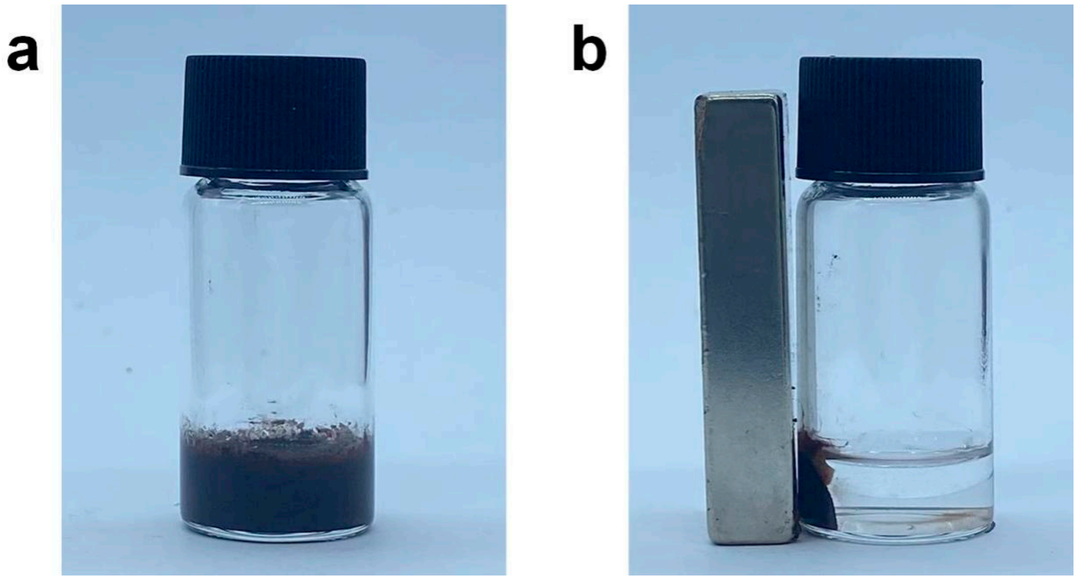

Figure S2. Photographs of (a) the MMJSP immobilized lipase dispersed in a solution and (b) the MMJSP immobilized lipase attracted by external magnetic fields.

Three-Phase Contact Angle Test

The solid particles on the surface of Pickering emulsions irreversibly adsorb at the oil-water interface, forming a dense interfacial film that prevents droplet aggregation. The closer the contact angle is to 90°, the stronger the anti-aggregation ability. As illustrated in Figure S3, the contact angle of the unmodified $Fe_3O_4@SiO_2$ particles is 25.6°, exhibiting strong hydrophilicity. After aldehyde modification, the contact angle increases to 79°, with enhanced hydrophobicity due to the benzene ring in terephthalaldehyde. After etching, the introduction of the hydrophobic long chain of N,N-dimethyldodecylamine further enhances the hydrophobicity, resulting in a contact angle of 87.2° for the Janus particles. This confirms the successful synthesis of Janus particles.

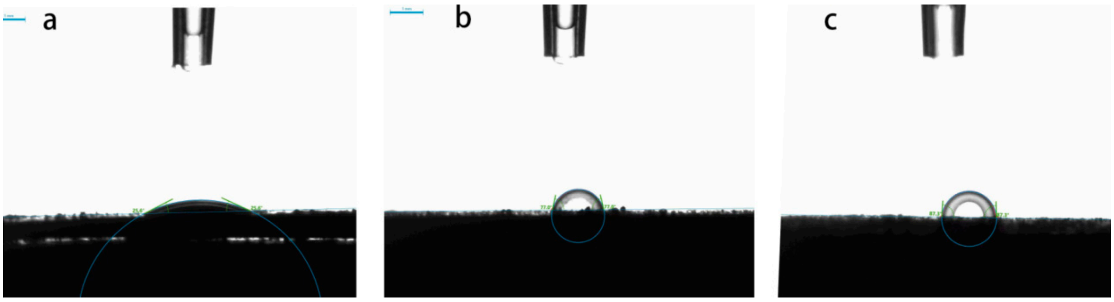

Figure S3. Contact angle of the material during preparation. (a) $Fe_3O_4@SiO_2$, (b) $Fe_3O_4@SiO_2@CHO$, and (c)Janus particles

Pickering Emulsion Stability Test

The establishment of the Pickering emulsion enzyme catalytic system relies on the continuous stability of the emulsion. As illustrated in Figure S4, the optical microscope

images of the emulsion stained with Nile Red after being left at room temperature for 1 hour, 3 hours, 6 hours, 18 hours, 24 hours, and 48 hours are shown in the figure. The stability of the emulsion droplet size distribution is also demonstrated. These results prove that the Pickering emulsion stabilized by Janus particles exhibits good stability.

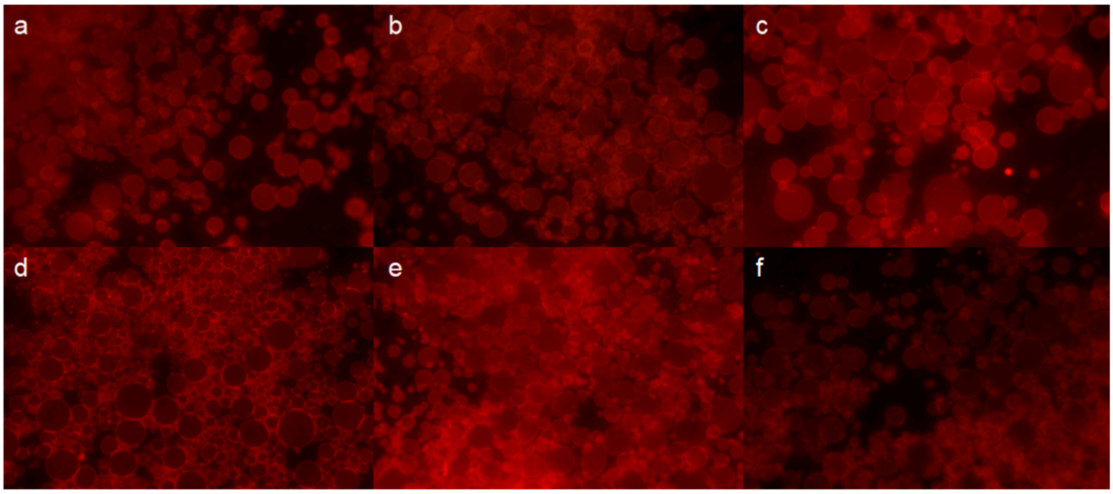

Figure S4. Microscopic images of Pickering emulsions formed by MMJSP-immobilized lipase with different storage times (a) 1h, (b) 3h, (c) 6h, (d) 18h, (e) 24h, and (f) 48h.

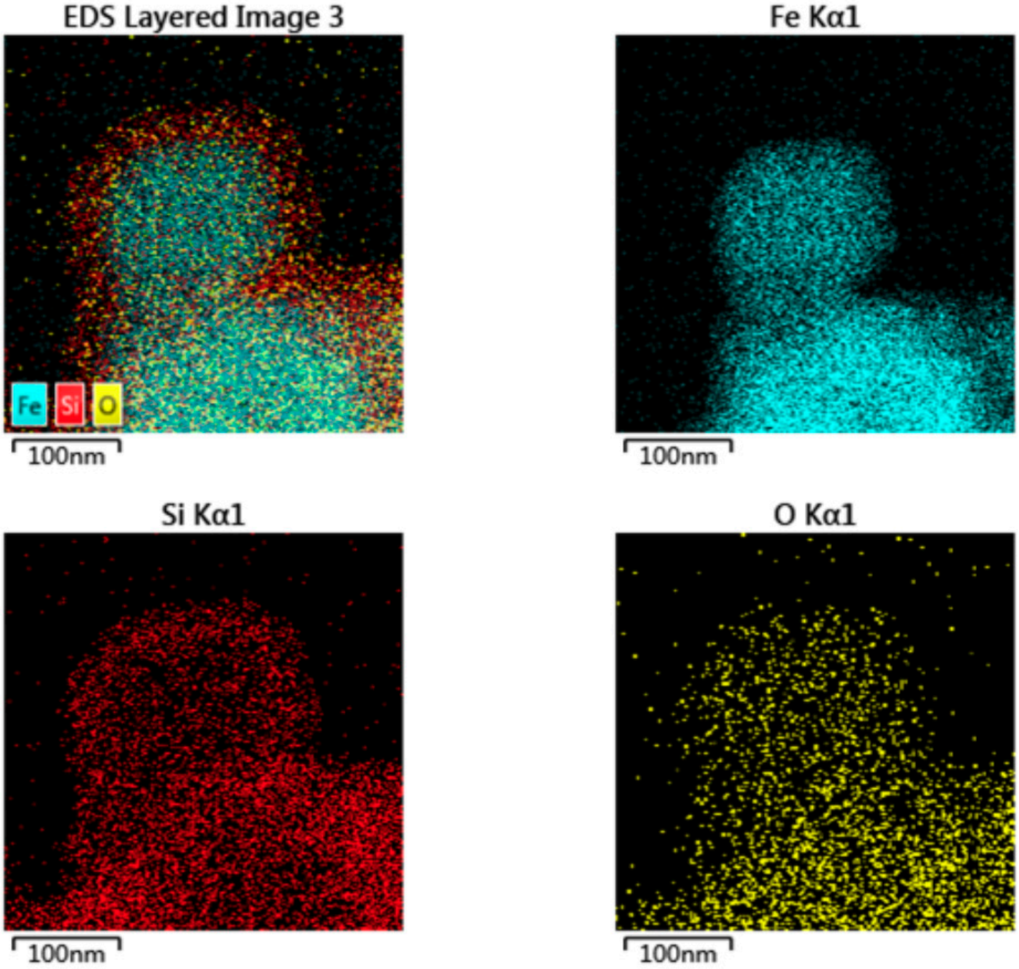

Figure S5. EDX mapping analysis of the MMJSP.

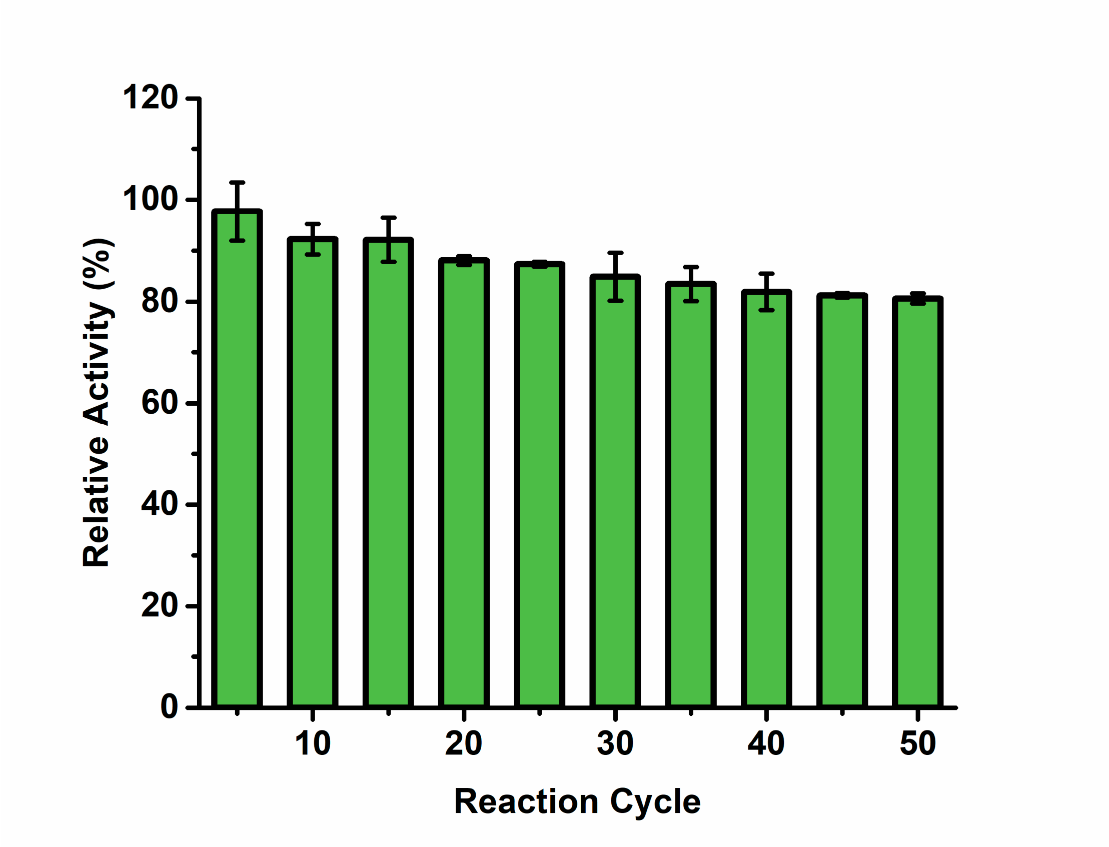

Figure S6. Enzyme activity of MMJN-immobilized lipase after 50 repeated uses in hydrolysis reactions.

Table S1. Kinetics parameters of the free and MMJN-immobilized lipase.

	Substrate	Km (mol/L·min)	Vmax (mol/L)
Free enzyme	pNPP	0.17×10^{-3}	0.41
Immobilized enzyme	pNPP	0.17×10^{-3}	0.44
